# Distribution model transferability for a wide-ranging species, the Gray Wolf

**DOI:** 10.1038/s41598-022-16121-6

**Published:** 2022-08-08

**Authors:** M. G. Gantchoff, D. E. Beyer, J. D. Erb, D. M. MacFarland, D. C. Norton, B. J. Roell, J. L. Price Tack, J. L. Belant

**Affiliations:** 1 Department of Environmental Biology, State University of New York College of Environmental Science and Forestry, Syracuse, NY 13210 USA; 2 Department of Fisheries and Wildlife, Michigan State University, East Lansing, MI 48824 USA; 3 Forest Wildlife Populations and Research Group, Minnesota Department of Natural Resources, Grand Rapids, MN 55744 USA; 4 Office of Applied Science, Wisconsin Department of Natural Resources, Rhinelander, WI 54501 USA; 5 Wildlife Division, Michigan Department of Natural Resources, Marquette, MI 49855 USA

**Keywords:** Conservation biology, Ecological modelling

## Abstract

Using existing data can be a reliable and cost-effective way to predict species distributions, and particularly useful for recovering or expanding species. We developed a current gray wolf (*Canis lupus*) distribution model for the western Great Lakes region, USA, and evaluated the spatial transferability of single-state models to the region. This study is the first assessment of transferability in a wide-ranging carnivore, as well as one of few developed for large spatial extents. We collected 3500 wolf locations from winter surveys in Minnesota (2017–2019), Wisconsin (2019–2020), and Michigan (2017–2020). We included 10 variables: proportion of natural cover, pastures, and crops; distance to natural cover, agriculture, developed land, and water; major and minor road density; and snowfall (1-km res.). We created a regional ensemble distribution by weight-averaging eight models based on their performance. We also developed single-state models, and estimated spatial transferability using two approaches: state cross-validation and extrapolation. We assessed performance by quantifying correlations, receiver operating characteristic curves (ROC), sensitivities, and two niche similarity indices. The regional area estimated to be most suitable for wolves during winter (threshold = maximum sensitivity/specificity) was 106,465 km^2^ (MN = 48,083 km^2^, WI = 27,757 km^2^, MI = 30,625 km^2^) and correctly predicted 88% of wolf locations analyzed. Increasing natural cover and distance to crops were consistently important for determining regional and single-state wolf distribution. Extrapolation (vs. cross-validation) produced results with the greatest performance metrics, and were most similar to the regional model, yet good internal performance was unrelated to greater extrapolation performance. Factors influencing species distributions are scale-dependent and can vary across areas due to behavioral plasticity. When extending inferences beyond the current occurrence of individuals, assessing variation in ecology such as habitat selection, as well as methodological factors including model performance, will be critical to avoid poor scientific interpretations and develop effective conservation applications. In particular, accurate distribution models for recovering or recovered carnivores can be used to develop plans for habitat management, quantify potential of unoccupied habitat, assess connectivity modeling, and mitigate conflict, facilitating long-term species persistence.

## Introduction

Understanding which factors limit species distributions is a foundational question in ecology and conservation, and species distribution models (SDMs) have become an important tool to map and predict species occurrences^1–5^. Use of SDMs has proliferated in the past several decades due to increasing accessibility and quantity of species occurrence data, the development of robust modeling algorithms (e.g.^6^), and the improvement of software and technological resources^7–9^. In addition, SDMs have been used successfully for on-the-ground conservation and management initiatives^10^. For example, information regarding species occurrence and distribution is critical for natural resource decision making, including International Union for Conservation of Nature plans^11^, invasive species management^12^, state wildlife action plans^13,14^, and conservation of endangered species^15,16^. In this context, researchers often use SDMs to understand species responses to different land uses and land covers, and spatial predictions can highlight priority locations to facilitate conservation initiatives.

Because natural resource management agencies and conservation organizations often have limited resources, using existing data to predict species distributions is an important line of research^17^. When species location data are unavailable or limited, researchers can estimate species distributions by transferring results across spatial and temporal extents and resolutions^18^. Studies on spatial transferability assess how well a model can be generalized to other areas^19,20^, such as evaluating niche opportunities for non-native species^21^ or species reintroduction assessments^22^. However, when models are overfitted to local conditions (i.e. when a model fits the calibration data too closely), performance on validation data and the spatial transferability of the model can be reduced^23^. Despite the importance of spatial transferability in distribution models, and the existence of studies designed to evaluate them (e.g.^19,24–26^), most are focused on species with marked ecological or climatic limits, or virtual species (i.e. simulations^27^), rather than wide-ranging or ecologically flexible species.

Evaluating transferability of SDMs is particularly important when assessing recovering species and species with expanding distributions, as it can provide forecasts of future distributions and provide insights into potential transferability of a model rather than proceeding under untested assumptions^28^. Specifically, SDMs for recovering or recovered carnivores can be used to develop plans for habitat management, quantify potential of unoccupied habitat, assess connectivity modeling, and mitigate conflict (e.g.^29–31^). Globally, large carnivores have experienced vast range contractions driven mostly by human persecution, loss and degradation of habitat, and prey depletion^32–34^. However, these declines can be reversed (e.g.^32^), and populations of several large carnivores are recovering, particularly in the northern hemisphere^34,35^. In North America, gray wolves (*Canis lupus*) have recolonized portions of their historic range after severe population declines in the mid-twentieth century^36,37^. Our objective was to develop a current wolf distribution model for the western Great Lakes region, expecting likelihood of occurrence to be positively influenced by increasing natural cover, and negatively influenced by increasing human disturbance^38,39^. In addition, we assessed SDM transferability by comparing model performance in making predictions within or outside the geographical domains of the model^1^. This study is the first assessment of SDM transferability in a behaviorally plastic and wide-ranging large carnivore, while also adding to a limited number of studies that assessed SDM transferability in any type of ecosystem at a regional (i.e. thousands of square kilometers) scale (e.g.^19,40,41^).

## Methods

### Species background

Wolves were nearly extirpated from the contiguous United States by the 1930s, mainly due to persecution to protect livestock, habitat loss, and prey declines^37^. By 1974, wolves were listed on the United States Endangered Species Act (16 U.S.C. 1531–1544, 87 Stat. 884). In January 2021, wolves in Michigan, Wisconsin, and Minnesota were removed from the United States list of Threatened and Endangered Species, however a federal judge restored protections on 10 February 2022, continuing a decades-long tug and pull between state and federal control of wolf management. Given the estimated population size (about 3800 individuals), the gray wolf population was considered recovered in the western Great Lakes region by the US Fish and Wildlife Service^37^, encompassing roughly two-thirds of the current total population in the contiguous United States^42^.

### Study area

Our study area comprised Minnesota, Wisconsin, and Michigan, USA. The northern portion of the study area is forest-dominated, with agriculture and human development predominant in the south. The area includes 44% forested lands, 31% cultivated crops, 6% pastures, and 7% developed lands; with lesser amounts of wetlands, shrubland, and herbaceous land covers^43^. Elevations ranges from 174 to 701 m above sea level. The area contains abundant lakes and streams, with most (86%) areas within 10 km of water. Human population density is low in northern portions of the study area and increases to > 100 people/km^2^ in southern portions.

### Wolf surveys

We collected wolf location data from winter surveys in Minnesota (2017–2018), Wisconsin (2019–2020), and Michigan (2017–2020). In Minnesota, the Minnesota Department of Natural Resources (MN DNR) mailed instructions to participants (i.e. trained natural resources staff at county, state, federal, and tribal agencies) and asked them to record locations and group size estimates of all wolves and wolf sign (e.g., track, scat) observed during normal work duties from November until snowmelt the following spring (about mid-May). Participants could record locations on forms or maps, but most data were entered by participants in a web-based GIS survey application. This database was combined with wolf observations and signs recorded during other wildlife surveys (e.g., carnivore scent station survey, furbearer winter track survey) coordinated by MN DNR.

In Wisconsin, the Wisconsin Department of Natural Resources (WI DNR) conducts wolf snow-track surveys every winter in areas of known or suspected wolf pack activity, with potential wolf range divided into 164 survey blocks to ensure comprehensive coverage. During winter 2019–2020, 158 blocks (96%) were sampled. Survey blocks were delineated to ensure an entire block could be surveyed in a day. Survey blocks average 500 km^2^ and are bordered by public roads, waterways, or state boundaries. Wisconsin Department of Natural Resources staff, Tribal biologists, and trained volunteers conduct the surveys 1–3 days after snowfalls and attempt to traverse most snow-covered roads in survey blocks. Trackers attempt to survey blocks at least 3 times (average 2.8 surveys per block) to identify the number of individuals in every pack.

In Michigan, the Michigan Department of Natural Resources (MI DNR), with assistance from the United States Department of Agriculture Wildlife Services (USDA WS), conducts wolf track surveys every other year during winter (December–March/April, e.g. 2018 survey was from December 2017 to April 2018), consisting of intensive and extensive searches of roads and trails by truck and snowmobile for wolf tracks and sign, to count the number of individuals in each pack. The MI DNR also attempts to capture and attach GPS collars to wolves in areas to be surveyed during the next survey period, to locate packs and spatially differentiate adjacent packs. The Upper Peninsula of Michigan, where wolves currently occur, is divided into 21 wolf survey units from which a random sample, stratified by historic wolf density, is drawn for each survey (targeting at least 50% of the Upper Peninsula to be surveyed). Michigan DNR and USDA WS staff are assigned to conduct track surveys in specific units, and surveys in adjacent units are coordinated to avoid duplicate counting of wolves.

To standardize the three datasets and decrease spatial autocorrelation, we filtered the data so no more than one location (randomly selected) occurred per 1-km^2^ pixel (i.e. the resolution of our covariates, see below). To further test if spatial autocorrelation influenced results, we additionally filtered data to no more than one location every 5 km. We compared modeling results for both datasets by assessing model performance (ROC and sensitivity, see below), and calculating the spatial correlation between resulting maps (using Band Collection Statistics in ESRI ArcMap 10.7).

### Distribution modeling

We considered 10 variables for our wolf distribution models: proportion of natural cover, proportion of pastures, proportion of cultivated crops, distance to natural cover, distance to agriculture, distance to developed land, distance to water, major road density, minor road density, and annual snowfall. All variables were resampled to 1-km resolution. For land cover, we used data from the National Land Cover Database 2016^43^. We regrouped the original land cover types into four categories: natural, cultivated crops, pastures, and developed cover. Natural cover included all non-developed covers (i.e. forest, grassland, shrubland, etc.), and developed cover included all four NLCD developed categories (i.e. open, low, high, and very high). We calculated proportion of land covers (natural, pasture, and crops) by aggregating the original 30-m resolution layer and calculating the proportion of each cover at the 1-km scale. We developed four distance to cover layers: distance to natural cover, distance to crops, distance to pastures, and distance to developed cover, at 1-km resolution. Using data from the National Hydrography Dataset (2016), we developed a distance to water layer (i.e. rivers and lakes). We created two road density layers using TIGER/Line Shapefiles (US Census Bureau): minor road density (county and local roads) and major road density (primary and secondary roads, e.g. highways and main arteries). Snowfall data (i.e. snow depth) was obtained from the National Weather Service National Snowfall Analysis (http://www.nohrsc.noaa.gov/snowfall) which estimates snowfall in the recent past by gathering several operational data sets into a unified analysis; we obtained the total annual winter snowfall from winters 2017–2018 to 2019–2020 and calculated the average across years. To compare the range of environmental values in each state, we created a variable range violin graph each state and the regional study area.

We created a correlation matrix using all GIS layers. We found strong correlation between distance to pastures and distance to crops (r = 0.82) as well as between proportion of natural cover and proportion of crops (r = − 0.78). To determine the best combination of variables, we ran all possible combinations for the regional model (never including two correlated variables in the same model) and chose the one with the greatest performance metrics (ROC and sensitivity, see below). Because models require background data (e.g. pseudo-absence points), we generated a randomly drawn sample of 10,000 background points from the study area, gave equal weight to presence and pseudo-absence points during modeling^44^. We used the entire study area to draw background points assuming all of it was available to wolves, as indicated by historical distribution^36,37^ and more recent data confirming sporadic occurrence outside areas snow track surveys were conducted (MN DNR, WI DNR, MI DNR, unpublished data).

We used an ensemble model approach to achieve more robust predictions^45^, combining 8 individual algorithms: random forest (RF), generalized boosted regression (GBM), Maximum entropy (MaxEnt), generalized linear model (GLM), generalized additive model (GAM), classification tree analysis (CTA), surface range envelop (SRE, also known as BIOCLIM), and flexible discriminant analysis (FDA) (algorithm details in Appendix S1). We assessed internal performance of individual models using threefold random cross-validation, with 80% of locations used as SDM training data and 20% as SDM testing data for each iteration. We evaluated models using the area under the curve of a receiver operating characteristic (ROC) plot, true skill statistics (TSS)^46^, and sensitivity scores represented as the ratio of presence sites correctly predicted over the number of positive sites in the sample^9^.

We created the ensemble model by weight-averaging all individual models proportionally to their performance evaluation metrics scores^9^, which resulted in a map representing continuous likelihood of presence. We quantified the influence of each variable in each individual model by permutation importance^9^, the greater the value of this metric, the more importance the predictor variable has on the model. To evaluate uncertainty, we created a committee averaging map, in which each individual model estimates if the species is present or absent in a pixel by transforming the continuous likelihood of presence to a yes (1) or no (0) binary response using an optimized threshold (maximum sensitivity and specificity). When the value of the committee averaging map is 1 or 0, it means that all models predicted presence (1) or absence (0), respectively. When the prediction approaches 0.5, about half the models identified the species as present. We used the biomod2 package^9^ in R v. 3.6.2 (R Core Team 2020) to develop individual models, ensembles, and committee consensus maps.

### Spatial transferability

Models are often sensitive to the spatial extent of the study area^47^ and background points strategy^48^, and dividing data into multiple geographic regions provides inference into how well models perform in unsampled regions^28^. We estimated spatial transferability using two complementary approaches: making predictions within (state cross-validation) or outside (extrapolation via restriction of background points) the geographical domains of the models. To perform a balanced comparison, for the spatial transferability analyses all three states had an equal number of points included in each model. Because Michigan had the lowest number of locations after filtering to 1-km resolution (478 locations), we randomly subsampled locations from the other two states to match that value.

For the cross-validation assessment, we used a spatially structured approach by creating three distribution models, each iteration using points from one state (i.e., MI, MN, and WI) as modeling locations, points from throughout the study area as background pseudo-absence locations, and wolf locations from the other two states as validation. For the extrapolation assessment, we similarly created three distribution models using points from only one state as modeling locations and the locations for the other two states as validation, however we restricted the background locations to occur only in the same state as the modeling locations (e.g., MI locations with MI only pseudoabsences). By doing this, we effectively created state-specific models, and then extrapolated the results to the other two unsampled states.

We quantified how well each single-state model (MI-crossvalidation, MN-crossvalidation, WI-crossvalidation, MI-extrapolation, MN-extrapolation, WI-extrapolation) predicted suitability for the study area by calculating Spearman correlation coefficients between all single-state and regional models, as well as ROC and sensitivity for the validation datasets. Additionally, we compared probability distributions with Schoener’s D and Hellinger’s I metrics^49,50^, which calculate niche similarity by comparing the estimates of suitability of each grid cell of the study area, and vary from 0 (no overlap) to 1 (complete overlap).

## Results

**Figure 1:**
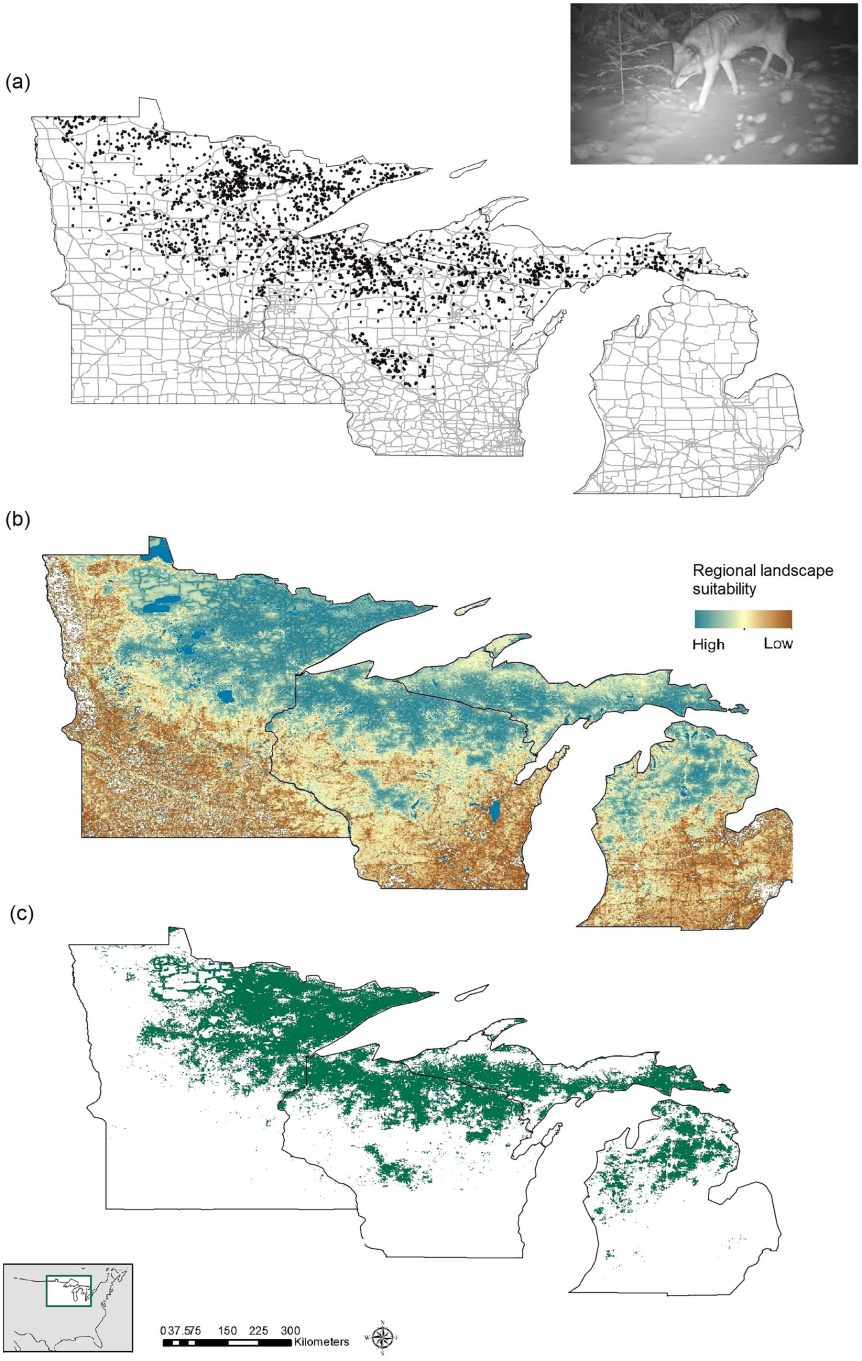
Wolf distribution in the western Great Lakes region, USA. (**a**) Wolf presence locations during 2017–2020 resampled to no more than one point per km^2^ with major roads shown as grey lines. (**b**) Regional landscape suitability (see “Methods” and Fig. 2). (**c**) Binary map indicating areas most suitable for wolves (suitability threshold 47.5%, see “Methods”). Maps developed with ArcMap 10.8.1 (desktop.arcgis.com). Wolf photograph: J. Belant, Global Wildlife Conservation Center.

We collected 3513 wolf locations: 594 in MI, 1597 in MN, and 1322 in WI. The 1-km filtering resulted in 2703 locations (MI 478, MN 1372, WI 853), and the 5-km filtering in 1315 locations (MI 241, MN 691, WI 383). Both filtered datasets resulted in ensemble models with high performance (ROC = 0.91 and sensitivity = 88% for 1 km, and ROC = 0.89 and sensitivity = 84% for 5 km). Due to almost identical performance and high spatial correlation between the two ensembles (98.2%), we considered the 1-km filtering process satisfactory and proceeded with analyses using this dataset (Fig. 1). Additionally, after testing all combinations of correlated variables (see “Methods”), the combination of proportion of natural cover and distance to crops had the best performance metrics (Appendix Table S1), therefore we excluded distance to pastures and proportion of crops from subsequent analyses.

The regional model (Fig. 1) had a ROC of 0.91. Variable permutation importance identified proportion of natural cover and distance to crops as having the greatest influence on regional wolf presence (Table 1). Likelihood of wolf presence increased with greater proportions of natural cover (~ 20% wolf likelihood at 0–20% natural cover vs. ~ 80% likelihood at 80–100% natural cover; Supplementary material Fig. S1) and greater distances from crops (20% wolf likelihood at 0 km from crops vs. 70–80% likelihood at ~ 1 km from crops). Less important variables included distance to developed cover (decreasing wolf likelihood with increasing distance), minor road density (greatest wolf likelihood at lower densities, with 50% of locations occurring at < 0.72 km/km^2^, and 90% at < 1.26 km/km^2^), and snowfall (wolf likelihood increasing to ~ 2.5 m snow depth then decreasing; Supplementary material Fig. S1). Using committee averaging, the greatest uncertainty in suitability predictions among individual models occurred in the periphery of the distribution (Supplementary material Fig. S2), with high consistency in the core distribution. Using the binary map (optimized threshold = 47.5% suitability), the estimated winter area most suitable for wolves was 106,465 km^2^ (MN = 48,083 km^2^, WI = 27,757 km^2^, MI = 30,625 km^2^, [MI upper peninsula = 18,812 km^2^, MI lower Peninsula = 11,813 km^2^]; Fig. 1), with wolf presence sensitivity of 88%, and pseudoabsence specificity of 75%.

**Table 1:** Variable permutation importance for individual models included in the regional model (see “Methods”) to estimate landscape suitability for wolves, western Great Lakes region, USA, 2017–2020. The greater the value, the more importance the predictor variable has on the model. Bold numbers indicate the top two performing covariates for each model.

	GLM	GBM	SRE	GAM	RF	FDA	CTA	MAXENT	Mean
Prop. of natural cover	**0.529**	**0.335**	**0.345**	**0.493**	**0.294**	**0.164**	**0.492**	**0.255**	**0.363**
Dist. to crops	**0.063**	**0.196**	**0.301**	**0.071**	**0.396**	**0.421**	**0.403**	**0.284**	**0.267**
Dist.to developed	0.026	0.027	0.095	0.042	0.164	0.037	0.083	0.045	0.065
Minor road density	0.050	0.020	0.137	0.042	0.154	0.020	0.052	0.036	0.064
Snowfall	0.008	0.020	0.055	0.021	0.220	0.051	0.034	0.086	0.062
Dist. to natural cover	0.041	0.000	0.132	0.033	0.017	0.000	0.000	0.017	0.030
Major road density	0.013	0.003	0.101	0.012	0.025	0.004	0.015	0.044	0.027
Distance to water	0.000	0.000	0.118	0.004	0.075	0.000	0.005	0.011	0.027
Prop. of pastures	0.010	0.001	0.092	0.011	0.030	0.000	0.033	0.019	0.025

**Figure 2:**
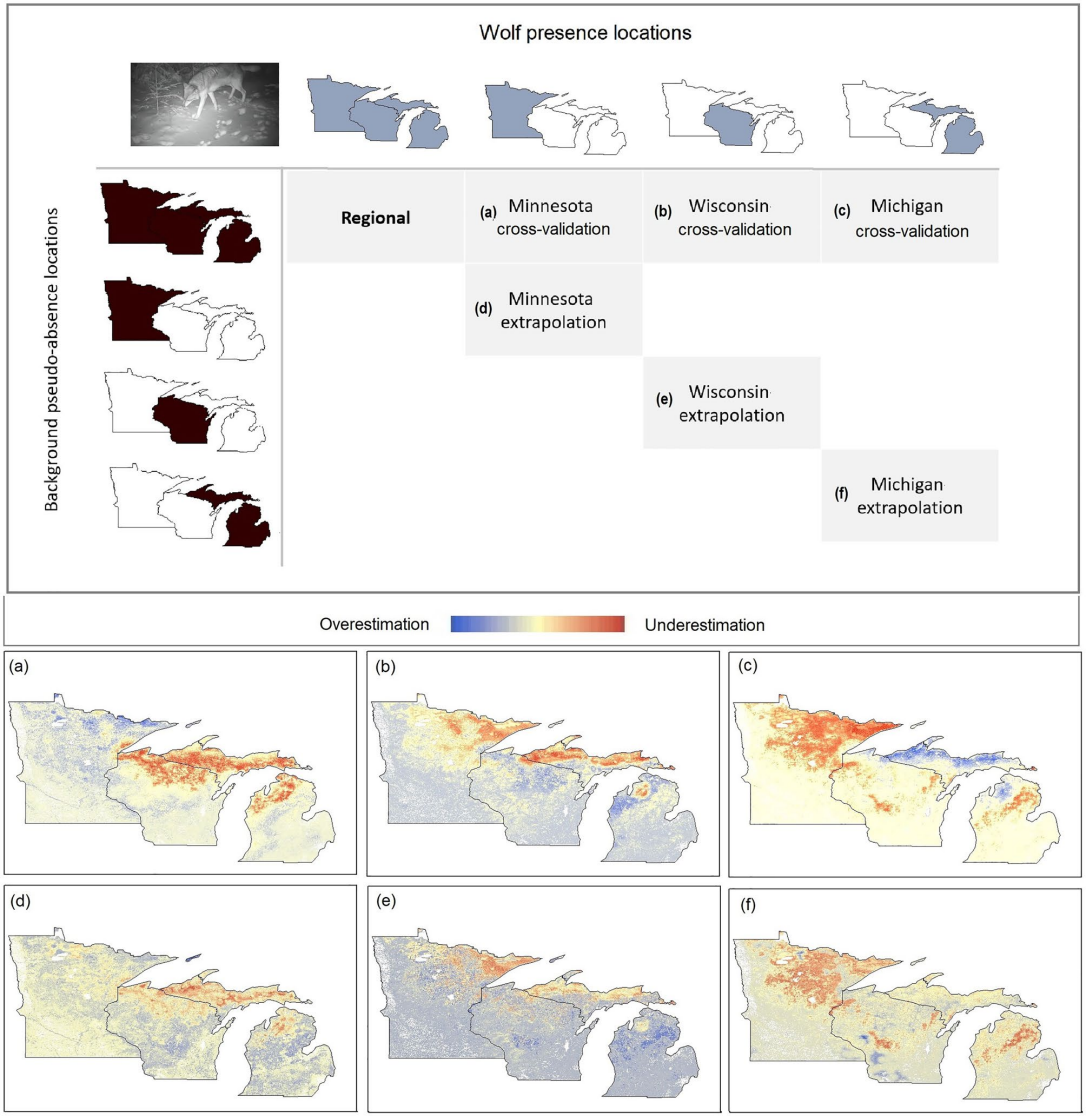
Top: Calibration (wolf presences) vs background (pseudo-absences) locations for the regional model, and cross-validation vs. extrapolation assessments. Bottom: Performance of each single-state model compared to the regional model (Fig. 1) in estimating landscape suitability for wolves, western Great Lakes region, USA, 2017–2020. Maps developed with ArcMap 10.8.1 (desktop.arcgis.com). Wolf photograph: J. Belant, Global Wildlife Conservation Center.

**Figure 3:**
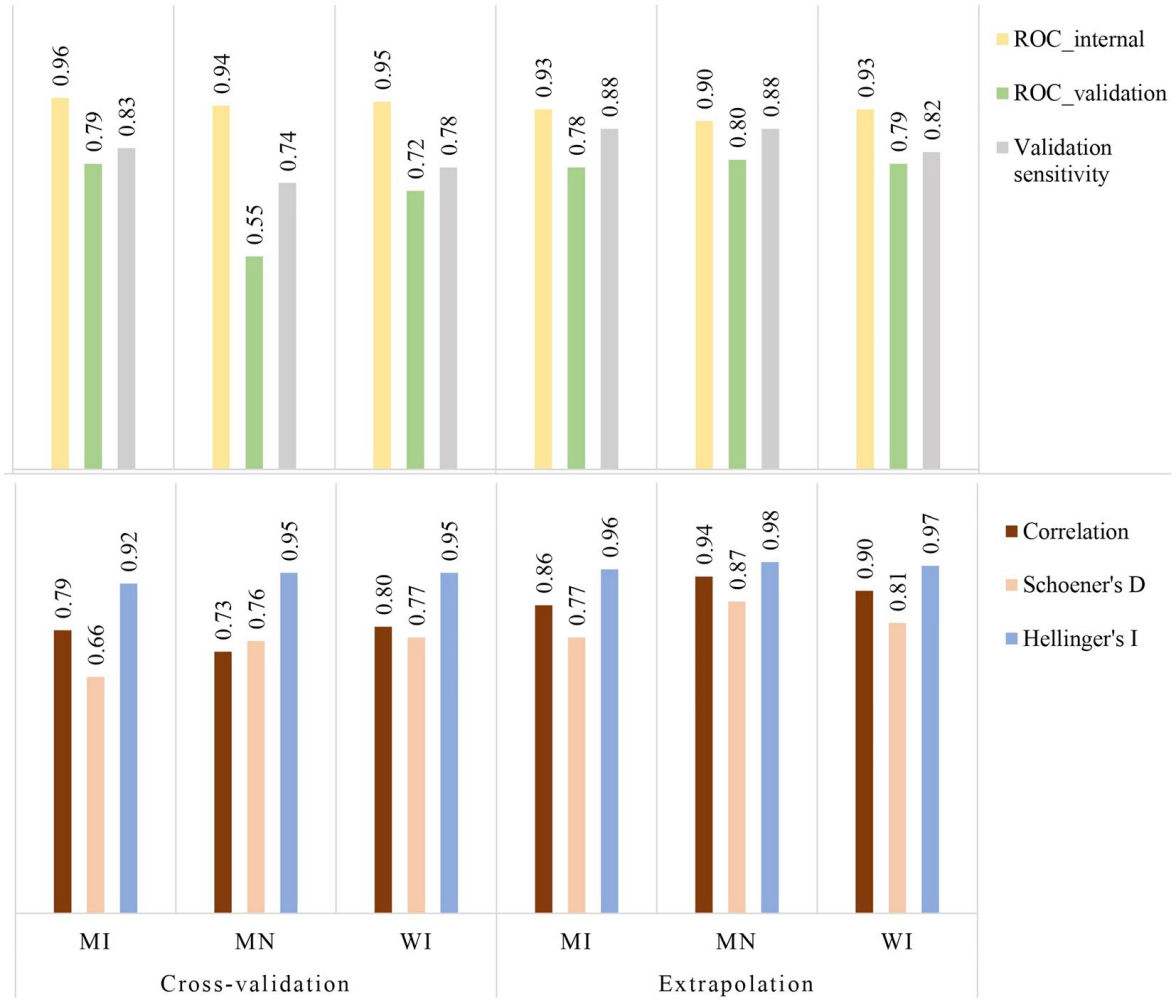
Performance metrics for the single-state distribution models (see “Methods”, Fig. 2) for wolves, western Great Lakes region, USA, 2017–2020. ROC_internal = area under the curve of an operator characteristic curve (ROC) for wolf calibration locations, ROC_validation = ROC for wolf validation locations, Validation sensitivity = proportion of correctly predicted wolf presence locations within the validation data, Correlation = Spearman’s r in relation to the regional model, Schoener’s D and Hellinger’s I = niche similarity metrics with the regional model (0 = no overlap, 1 = complete overlap, see “Methods”), *MI* Michigan, *MN* Minnesota, and *WI* Wisconsin.

Compared to the regional model, each single-state cross-validation model over- or underestimated suitability in different parts of the study area (Fig. 2), with overestimation often surrounding the presence locations. The WI-crossvalidation model had the greatest correlation with the regional model and the highest sensitivity for the validation points (0.74 and 78% respectively), followed by MN, then MI (Fig. 3). Internal ROC values were overall high (0.93–0.96), however the validation ROC values were markedly lower (Fig. 3), being greatest for MI and WI, followed by MN.

Comparisons between the regional model and each single-state extrapolation model also indicated over- or underestimation of suitability in different parts of the study area, though not as strongly as the cross-validation models (Figs. 2, 3). The correlation values with the regional model and validation sensitivity percentages were high; best performing was MN (0.92 and 88% respectively), followed by WI and MI (Fig. 3). Internal ROC values were again overall high (0.90–0.93), with validation ROC values lower (Fig. 3), but similar for the three states (0.78–0.80).

**Figure 4:**
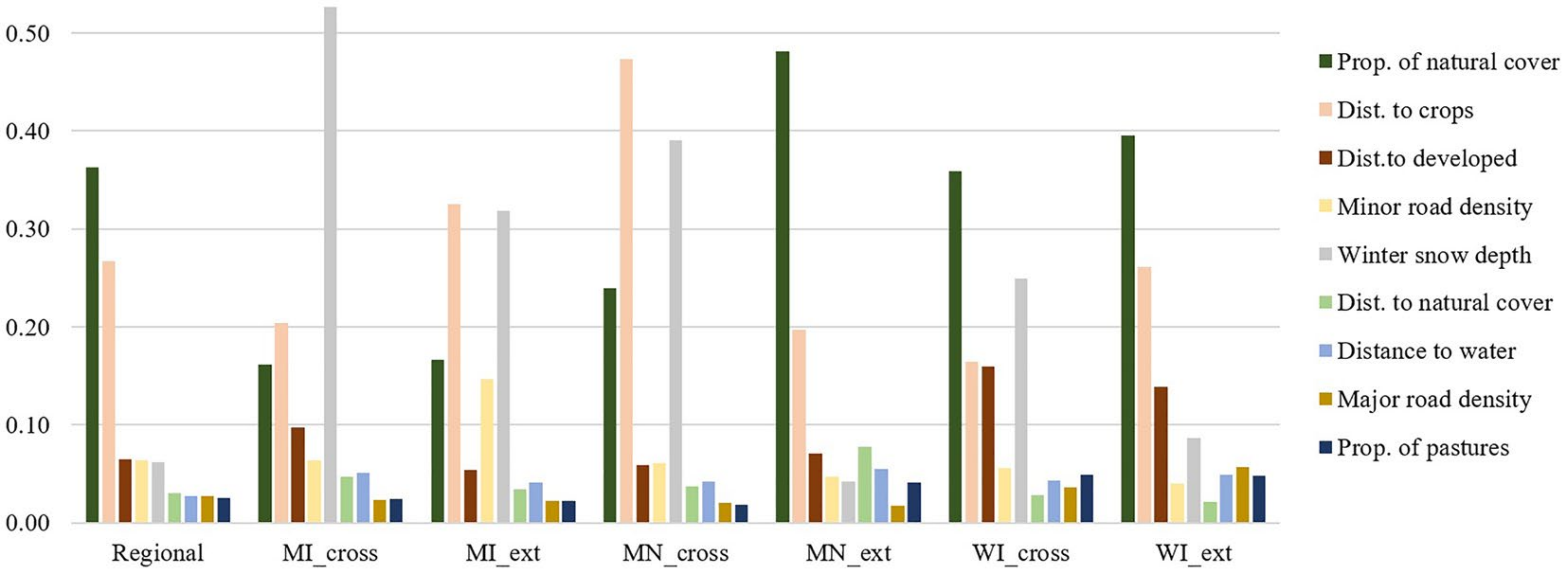
Average variable permutation importance (8 models, see “Methods”) for wolf distribution models, western Great Lakes region, USA, 2017–2020. Shown are the regional model and each single-state cross-validation and extrapolation model (see Fig. 2). *MI* Michigan, *MN* Minnesota, *WI* Wisconsin, ‘*cross*’ cross-validation, and ‘*ext*’ extrapolation.

The comparison of cross-validation and extrapolation results indicated that overall, the extrapolation models performed more similarly to the regional model, with greater correlation with the regional model, higher sensitivity for validation points, greater validation ROC values, and greater Schoener’s D and Hellinger’s I metrics. However, for internal prediction (within the spatial extent of presence locations used), the single-state cross-validation assessment resulted in marginally greater mean ROC values (0.95 vs. 0.92). The variable permutation importance of single-state models revealed the most influential variables were similar, with distance to crops and proportion of natural cover (the two most influential for the regional model) always among the top three variables (Fig. 4). However, snowfall was also sometimes within the top 3 variables, with widely different ranking across states. The variable range violin graph revealed that the available environmental gradient varied among states, with only two variables (i.e. proportion of natural and proportion of pastures) having similar ranges in all three states (Supplementary material Fig. S3). Wolves overall did not appear to select for or against any specific distance to developed land, using them as available throughout the region (Supplementary material Fig. S3).

## Discussion

We developed a current wolf winter distribution model for the western Great Lakes region and evaluated model transferability comparing single-state model predictions to the regional model. Single-state models with restricted background points performed slightly better than the regional model at describing wolf distribution within the geographic extent of their presence locations, likely because local models are able to detect smaller-scale variations more effectively than global models that incorporate greater heterogeneity in environment–species relationships^19,41^. In contrast to a previous study^40^, our spatial transferability assessment indicated that extrapolation produced results most similar to the regional model, as well as having better performance in relation to the validation data. Proportion of natural cover and distance to crops were the most important covariates determining regional wolf distribution, and single-state models also consistently indicated crops and natural cover among the three most influential variables.

Among states, we expected good internal performance (ROC) to be correlated with extrapolation performance^51^, but we found no such relationship between internal ROC and validation ROC. Similarly, a study in the Iberian Peninsula found that better internal performance metrics did not correspond with extrapolation success^19^. We observed variable extrapolation performance among states, likely a result of environmental differences or biotic factors such as area-specific species interactions^20^. From a modeling perspective, the differences in the variable extent within each state is expected to influence their extrapolation performance. For example, MN is the only state that envelops the complete extent of values for distance to crops (see Supplementary material, Fig. S3), a key variable for the regional wolf distribution model and possibly resulting in the better performance we observed for the MN-extrapolation model, and the poor performance when extrapolating to MN from the other two states. Barbosa and coauthors^19^ concluded that the analyzed range of values of the predictors is possibly more important than other factors, such as having a dataset free of false absences.

The regional model results suggested that distance to crops and proportion of natural cover were the most influential covariates explaining wolf distribution across the Great Lakes region. Previous studies in this area also found that wolves avoid agricultural land^52^, occur in forest cover^52–54^ and select for natural areas while avoiding human-modified covers including pastures, hayfields, and farms^55^. Wolves in Europe behaved similarly, selecting wild areas far from human disturbance^39^. Snowfall had minor importance at the regional level, but was more influential at the state level, particularly in Michigan, likely because it is the state with the greatest snowfall range variation, and wolves occur in some of the northernmost areas within the state. At finer spatial scales wolves seem to select areas with less snow, potentially in response to prey distributions^56^ or human activities^57^, which suggests that snowfall has a scale-dependent effect on winter wolf distribution.

All models predicted the northern half of the Lower Peninsula of Michigan to have suitable areas for wolves, though wolves are absent. The Lower Peninsula is the last major area of the western Great Lakes region with potential habitat where a breeding population of wolves are not established^58^. Gray wolves have only rarely been sighted in the Lower Peninsula in the past 15 years, even though wolves could cross the Straits of Mackinac (separating the Lower from the Upper Peninsula) during winters with adequate ice formation^59^. Habitat-based density estimates have calculated the potential for 40–105^60^ or 52–63 wolves^59^ in the Lower Peninsula. However, greater proportions of livestock-based agriculture in the Lower Peninsula, as well as greater road and human densities, may result in increased challenges such as human-caused wolf mortalities and human-wolf conflicts.

Persistence of large carnivores in human-modified landscapes is facilitated by their behavioral plasticity, which allows them to adapt to human activity through variable spatiotemporal patterns of habitat selection that facilitate human avoidance while supplying resources for persistence (e.g. prey, resting sites, etc.). In particular, road density has been identified as a major determinant of wolf presence, with wolf probability very low in areas with road densities exceeding 0.7 km/km^2^^54,59,60,62^. However, half of the wolf locations in our study area occurred above this threshold (90% occurred below 1.26 km/km^2^), which could in part be influenced by our survey methods. Wolf responses to low-traffic roads are context-dependent, conditional on ease of travel, human settlements, time of day, prey densities, mortality risks, and seasons (e.g.^63–67^). Behavioral responses of wolves to anthropogenic disturbance can also vary due to internal factors, such as behavioral states and social affiliations^61^. Nonetheless, suitable land covers (i.e. forests and shrublands) are consistently selected for, suggesting some habitat selection patterns will persist regardless of context (e.g. selection of natural cover). Although behavioral plasticity might facilitate wildlife occurrence, it might not be enough to ensure long-term population viability and persistence in areas with decreasing habitat quality and availability.

When species absence data are unavailable, SDMs (such as in this study) use pseudo-absences, and the environmental span of the background from which pseudo-absences are drawn has important ramifications for predictions and performance of SDMs^68–70^. Defining the spatial extent of pseudo-absences can be subjective (except for populations limited by geographical barriers), and different strategies have been proposed to improve the selection of an appropriate dataset (e.g. random, environmental exclusion, minimum–maximum distance), with some distribution modeling techniques including regression being more affected than others (i.e. machine learning, classification trees; see^44^). Specifically, limiting the maximum distance of background points may improve sensitivity performance^44,70^. In agreement, we found that for each single-state model, though restricting background points to the same state as the calibration data had no clear effect in internal performance, it always increased validation sensitivity values, indicating improved discrimination ability. Despite being beyond the scope of this study, we highlight how the background choice can influence SDM results, and encourage further exploration of this topic (see^68,70^).

## Conclusions

While using existing data to predict species patterns for areas with limited information is a valuable and relevant research topic^17^, transferring model results into unsampled regions is more complex than simply filling gaps within a landscape^26^. We present a first assessment of the current distribution and spatial transferability for a flexible and wide-ranging large carnivore, finding that extrapolation had better predictive power into unsampled states, and that among states, good internal performance did not ensure extrapolation success. Consideration of these limitations can help develop better spatially-explicit models of conservation priority areas, which are becoming increasingly important^71,72^. Assessing spatial transferability performance is key when assessing expanding and recovering species and can identify concerns with extending inferences beyond the current occurrence of individuals. Matching distribution models to the needs of particular objectives^73^, and assessing variation in predictive power, internal performance, as well as ecological and behavioral inferences, will continue to be critical to avoid poor scientific interpretations and develop appropriate conservation and management applications.

## Supplementary Information

Supplementary Information.

## Data Availability

The data that support the findings of this study are available from the Michigan, Minnesota, and Wisconsin Departments of Natural Resources. Legal restrictions apply to the availability of these data, which were used under agreement for the current study, and so are not publicly available. Data should be requested from each natural state agency (see author list for reference).
