# Supplementary material for: Distribution model transferability for a wide-ranging species, the Gray Wolf
Source: Sci Rep. 2022 Aug 8;12:13556. doi: 10.1038/s41598-022-16121-6 (PMC9359985; doi:10.1038/s41598-022-16121-6)
Supplement: Supplementary file 1 — Supplementary Information. [file 41598_2022_16121_MOESM1_ESM.docx]

Supplementary material

**Gantchoff MG, Beyer, DE Jr., Erb J, MacFarland D, Norton D, Price Tack J, Roell B, Belant JL. Distribution model transferability for a wide-ranging species, the Gray Wolf.**

*Appendix S1. Modeling techniques*

**GLM - Generalized Linear Models.**

A less restrictive form than classic multiple regressions by providing error distributions for the dependent variable other than normal and non-constant variance functions. To select for the most parsimonious model, the algorithm uses an automatic stepwise model selection using Akaike Information Criterion (AIC). Key reference: McCullagh, P. and Nelder, J.A. (1989) Generalized linear models Chapman and Hall.

**GAM - Generalized Additive Models**

General additive models are designed to capitalize on the strengths of GLMs without requiring a response curve shape or specific parametric response function. They use a class of equations called "smoothers" that attempt to generalize data into smooth curves by fitting subsections of the data. BIOMOD uses a cubic spline smoother and automatic stepwise model selection with AIC. Key reference: Hastie, T.J. and Tibshirani, R. (1990) Generalized additive models Chapman and Hall, London.

**CTA - Classification Tree Analysis**

This method consists of recursive partitions of the dimensional space defined by the predictors into groups that are as homogeneous as possible in terms of response. A tree is built by repeatedly splitting the data, defined by a simple rule based on a single explanatory variable. The best tree is a trade-off between a high decrease of deviance and the smallest number of leaves. Key reference: Breiman, L., Friedman, J.H., Olshen, R.A., and Stone, C.J. (1984) Classication and regression trees. Chapman and Hall, New York.

**GBM - Generalized Boosting Models (or boosting regression trees)**

Boosting methods fit a large number of relatively simple models whose predictions are combined to give more robust estimates of the response. In BIOMOD, each model is a simple classification or regression tree, that is, a rule-based classifier that consists of recursive partitions of the dimensional space defined by the predictors into groups that are as homogeneous as possible in terms of response. From this, the GBM uses an iterative method to develop a final model by progressively adding trees, while re-weighing the data to emphasize cases poorly predicted by the previous trees.

Key references: Friedman, J.H. (2001) Greedy function approximation: a gradient boosting machine. Annals of Statistics, 29, 1189-1232.

Friedman, J.H., Hastie, T.J., and Tibshirani, R. (2000) Additive logistic regression: a statistical 84 view of boosting. Annals of Statistics, 28, 337-374.

Ridgeway, G. (1999) The state of boosting. Computing Science and Statistics, 31, 172-181.

**Random Forest - Breiman and Cutler's random forest for classification and regression**

Random Forests develop many classification trees. To classify a new object from an input vector, put the input vector down each of the trees in the forest. Each tree gives a classification, and the tree "votes" for that class. The forest chooses the classification having the most votes (over all the trees in the forest). It is implemented into the "random- Forest" library programmed by Andy Liaw and Matthew Wiener. R-BIOMOD uses 500 trees by default and extracts the importance of each selected variable. Key Reference: Breiman, L. (2001), Random Forests. Machine Learning 45, 5-32.

**SRE - Surface Range Envelops**

This is a simple surface range envelop, similar to BioClim. The envelop is defined by identifying maximum and minimum values for each input variable from the set of sites containing an observed species' presence. Any site with all variables falling between these maximum and minimum limits is included within the range. Key reference: Busby JR (1991) BIOCLIM - a bioclimate analysis and prediction system. In: Margules CR, Austin MP, editors. Nature conservation: cost effective biological surveys and data analysis. Canberra, Australia: CSIRO. pp. 64-68.

**Flexible Discriminant Analysis (FDA)**

A classification model based on a mixture of linear regression models, which uses optimal scoring to transform the response variable such that the data are in a better form for linear separation, and multiple adaptive regression splines are used to generate the discriminant surface.

Key reference: Hastie, T., Tibshirani, R., & Buja, A. (1994). Flexible discriminant analysis by optimal scoring. Journal of the American statistical association, 89, 1255-1270.

**Maximum entropy (MaxEnt)**

MaxEnt estimates a target distribution by finding the distribution of maximum entropy (i.e., that is closest to uniform), subject to the constraint that the expected value of each feature under this estimated distribution matches its empirical average. This approach is equivalent to finding the maximum likelihood Gibbs distribution (i.e., distribution that is exponential in a linear combination of the features).

Key references: Phillips, S. J., Dudík, M., & Schapire, R. E. (2004). A maximum entropy approach to species distribution modeling. In Proceedings of the twenty-first international conference on Machine learning.

Phillips, S. J., Anderson, R. P., Dudík, M., Schapire, R. E., & Blair, M. E. (2017). Opening the black box: An open‐source release of Maxent. Ecography, 40, 887-893.

Some example tutorials and code for *biomod2* can be found at:

http://www.will.chez-alice.fr/pdf/BiomodTutorial.pdf

https://github.com/biomodhub/biomod2-tutorial-materialTable S1. Performance metrics for all potential combinations of correlated explanatory variables (see methods) for wolf species distribution models, western Great Lakes region, USA, 2017–2020. ROC = Area under the curve for a receiver operating characteristic curve, Sensitivity = percentage of correctly predicted presences from internal validation.

| Variable combination | ROC | Sensitivity (%) |
| --- | --- | --- |
| Prop of natural cover + Dist. to crops | 0.90 | 90 |
| Prop of natural cover + Dist. to pastures | 0.88 | 88 |
| Prop of crops + Dist. to crops | 0.89 | 86 |
| Prop of crops + Dist. to pastures | 0.88 | 88 |

Table S2. Correlation values among wolf distribution models (see methods, Figure 2), western Great Lakes region, USA, 2017–2020. MI = Michigan, MN = Minnesota, WI = Wisconsin, cross = cross-validation, and ext = extrapolation.

|  | MI_ext | MI_cross | MN_ext | MN_cross | WI_ext | WI_cross |
| --- | --- | --- | --- | --- | --- | --- |
| MI_cross | 0.89 |  |  |  |  |  |
| MN_ext | 0.69 |  |  |  |  |  |
| MN_cross |  | -0.02 | 0.72 |  |  |  |
| WI_ext | 0.78 |  | 0.78 |  |  |  |
| WI_cross |  | 0.65 |  | 0.23 | 0.87 |  |
| Regional | 0.81 | 0.64 | 0.92 | 0.70 | 0.85 | 0.74 |


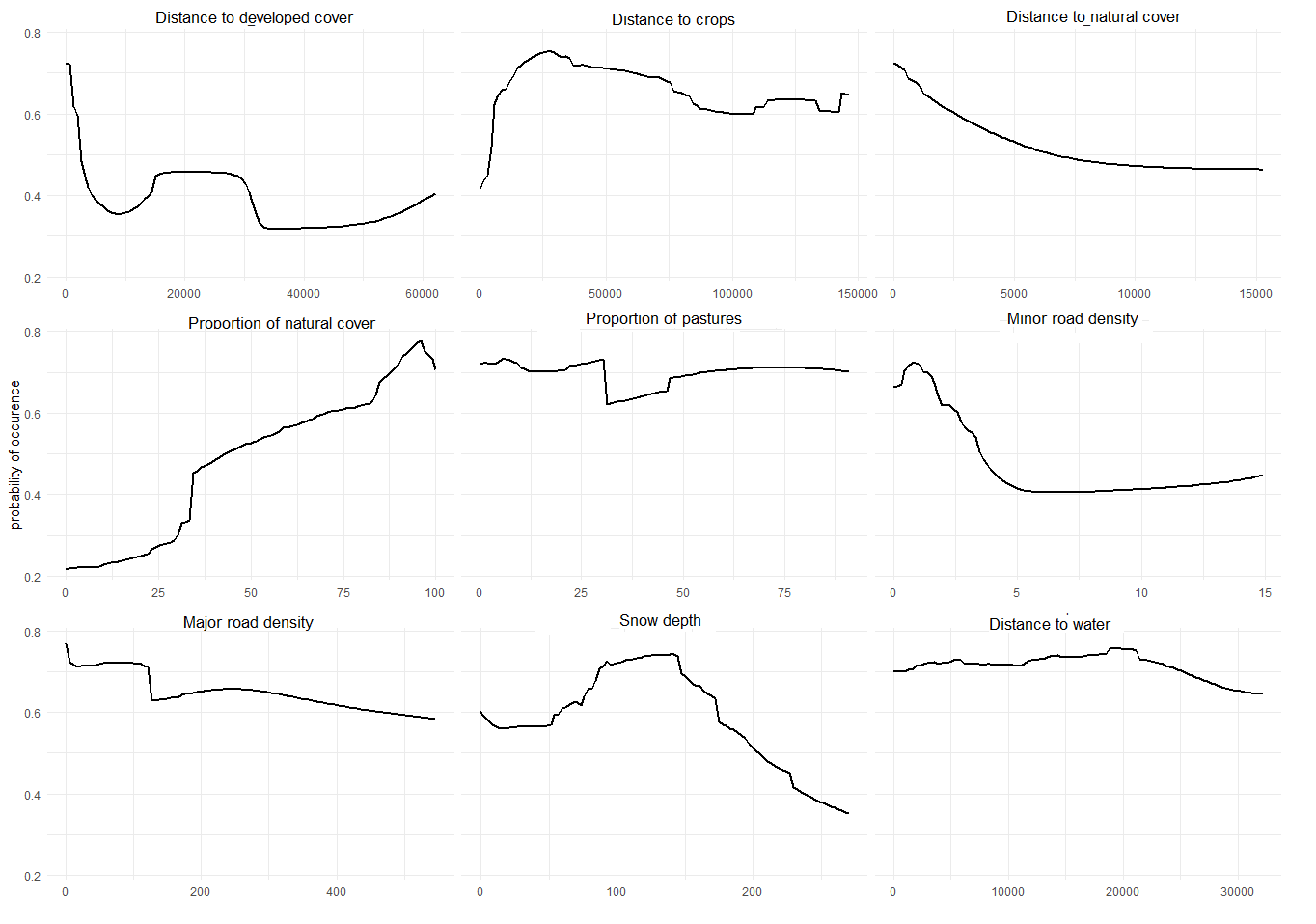


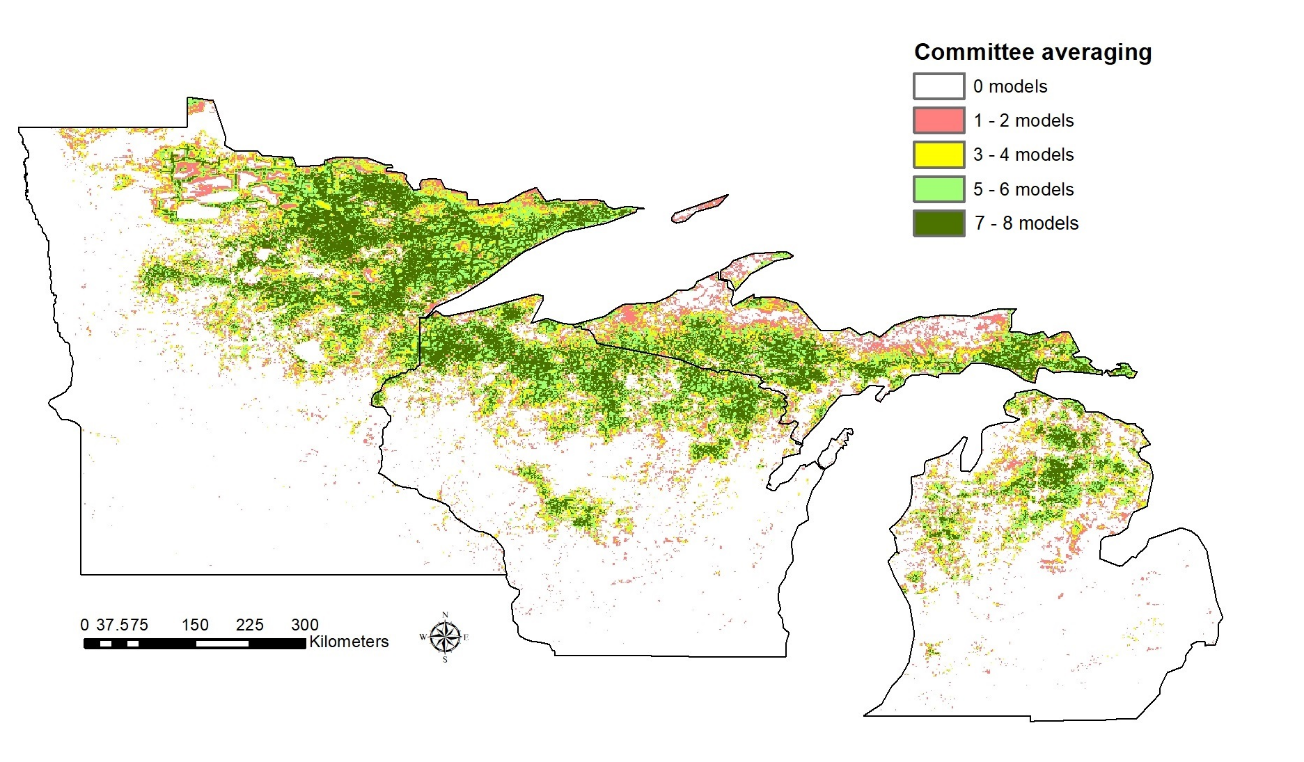
Figure S1. Variable response curves (R package *biomod2* v. 3.4.6) for the regional model suitability model for wolves, western Great Lakes region, USA, 2017–2020.

Figure S2. Committee averaging map (R package *biomod2* v. 3.4.6, ArcMap v. 10.7) indicating number of individual models (see methods) that identify an area as suitable for wolves, western Great Lakes region, USA, 2017–2020. Map made with ArcMap 10.8.1 (desktop.arcgis.com).


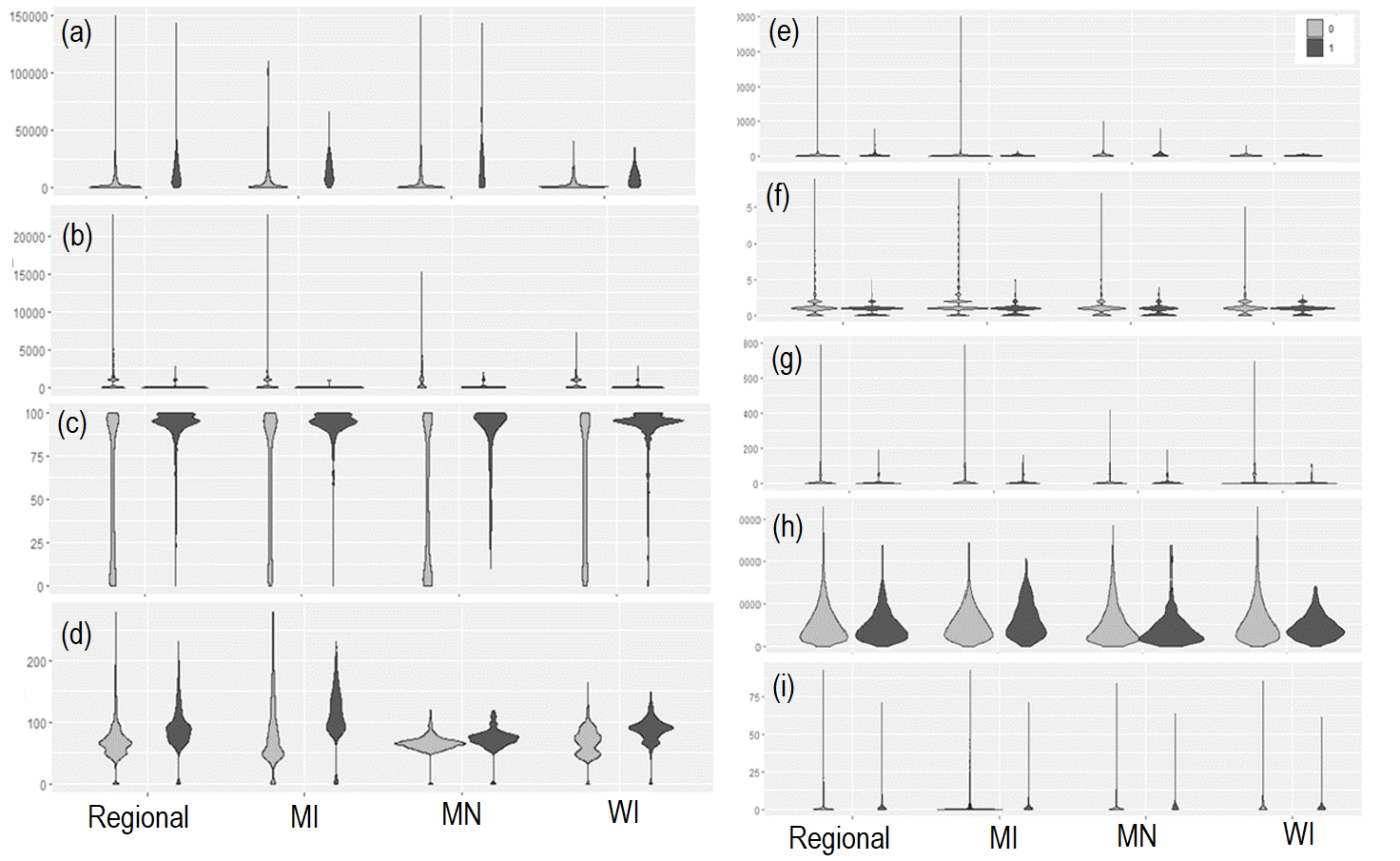


Figure S3. Violin plots for background points (light grey) and wolf locations (dark grey) for each state (MI = Michigan, MN = Minnesota, WI = Wisconsin) and Regional (the 3 states combined), USA. (a) distance to crops, (b) distance to natural cover, (c) proportion of natural cover, (d) snowfall, (e) distance to developed cover, (f) minor road density, (g) major road density, (h) distance to water, and (i) Proportion of pastures.
